# In Vitro and In Vivo Efficacy of Romidepsin Alone and in Addition to Standard of Care for Treatment of Ewing Sarcoma

**DOI:** 10.3390/cancers17244018

**Published:** 2025-12-17

**Authors:** Kaitlyn H. Smith, Erin M. Trovillion, Kimberly Q. McKinney, Poornima Gourabathini, Kenzie Wells, Divya Gandra, Chloe Sholler, Ingrid Votruba, Javier Oesterheld, Giselle L. Saulnier Sholler

**Affiliations:** 1 Atrium Health Wake Forest Baptist Comprehensive Cancer Center, Charlotte, NC 28203, USA; 2 Levine Children’s Hospital, Charlotte, NC 28203, USA; 3 Penn State Health Children’s Hospital, Hershey, PA 17033, USA

**Keywords:** Ewing sarcoma, pediatric oncology, HDAC inhibition

## Abstract

**Background:** Ewing sarcoma (ES) is an aggressive malignancy and there is an unmet need for more effective treatment options for patients. Histone deacetylases (HDACs) have been shown to be involved in ES tumorigenesis and HDAC inhibitors have been investigated in the context of ES. Our objective for this study was to investigate the efficacy and mechanism of action of HDAC inhibition in vitro and in vivo in ES models, alone and in combination with standard of care therapies. **Methods/Results:** HDAC inhibitors were tested for in vitro efficacy against ES cell lines and romidepsin was found to be most effective. The mechanistic changes induced by romidepsin were investigated by Western blotting and proteins involved in cell cycle progression and DNA damage repair were found to be repressed. In vitro we identified that romidepsin synergizes with doxorubicin and etoposide and that it increases the efficacy of the standard of care combinations VDC/IE. Further, the combination treatments lead to an increase in caspase 3/7 cleavage, a decrease in DNA damage repair proteins, and an accumulation of DNA damage. In vivo, the combination of romidepsin and ifosfamide/etoposide (IE) leads to a significant decrease in tumor volume compared to that of IE alone. **Conclusions:** Our data indicates that romidepsin improves efficacy of chemotherapeutic agents in vitro and leads to a decreased tumor volume in vivo, suggesting that the addition of romidepsin may improve upfront treatment in ES patients.

## Simple Summary

Survival rates for Ewing sarcoma patients have not improved in several decades, highlighting the importance of identifying new and more effective treatment options. We have identified that the HDAC inhibitor romidepsin improves efficacy of standard of care chemotherapy combinations. When combined with standard of care agents in vitro, romidepsin acts synergistically and leads to an accumulation of DNA damage. Further, romidepsin improves efficacy of standard of care combinations. In mouse xenograft models, the addition of romidepsin to the ifosfamide/etoposide standard of care chemotherapy combination led to a decrease in tumor burden compared to that of the chemotherapy alone. This data suggests that the addition of romidepsin may improve efficacy of standard of care in Ewing sarcoma.

## 1. Introduction

Ewing sarcoma (ES) is an aggressive malignancy which arises from the bone or soft tissue and most commonly occurs in children and young adults. Current treatment options, including chemotherapy, surgery, and radiotherapy, have led to a 5-year survival rate of approximately 70% for localized disease and less than 30% for patients with metastatic or relapsed disease [1,2]. Further, patients who relapse within 2 years of initial diagnosis have a 5-year survival rate of <10%. These poor survival rates highlight the unmet need of newer and more effective treatment options for ES patients [3,4].

The chromosomal translocation leading to the EWS:FLI1 fusion oncoprotein is seen in 85% of ES tumors [5]. This fusion protein is a transcription factor which leads to induction of several genes involved in cell proliferation and survival and which promote tumorigenesis [6]. There have been efforts investigating the efficacy of targeted agents, including those targeting poly (ADP-ribose) polymerase (PARP) [7], Aurora kinase A (AURKA) [8], cyclin-dependent kinase 4 and 6 (CDK4/6) [9], and the EWS:FLI1 fusion protein itself [10,11], though none of these agents have been promising clinically.

Class 1 histone deacetylases (HDACs) have been shown to contribute to tumorigenesis and metastasis, promote cell proliferation, and inhibit apoptosis [12,13,14]. In the context of ES, high levels of class 1 HDACs have been associated with decreased survival, and HDAC2 specifically has been shown to be overexpressed in a panel of ES patient tumors [15,16]. The role of HDACs in many cancer types has led to the development and approval of several HDAC inhibitors for cancer treatment, including vorinostat, panobinostat, belinostat, and romidepsin. Vorinostat, Panobinostat, and belinostat are nonselective pan-HDAC inhibitors, while romidepsin is a selective HDAC1/2 inhibitor [17].

The current chemotherapeutic agents used as standard of care in the United States for ES include vincristine, doxorubicin, cyclophosphamide, ifosfamide, and etoposide and there has been a lack of new effective targeted treatment options identified. The poor survival rates, especially in the metastatic or relapse patient population, highlight the need for new therapeutic options. Our group, as well as others, have previously shown that HDAC inhibition by panobinostat is toxic to ES cells and that HDAC inhibitors synergize with standard of care drugs in vitro [15,16], though the addition of HDAC inhibition, specifically class 1 inhibition, to the standard of care chemotherapy regimens has not been fully investigated in vivo.

Thus, this study was designed to further investigate the mechanism of HDAC inhibition and evaluate the efficacy in vitro and in vivo both alone and in combination with chemotherapy combinations. Additionally, we investigated the mechanism of synergy between HDAC inhibition and SOC. The overall goal of this study is to provide further evidence of the efficacy of HDAC inhibition in ES and to identify a potential treatment combination, which could lead to new treatment options for ES patients.

## 2. Materials and Methods

### 2.1. Cell Lines and Culture

All patient derived cell lines were obtained from the Beat Childhood Cancer Consortium and maintained as previously described [16]. A673 cells were obtained from ATCC and cultured in high glucose DMEM media (ThermoFisher Scientific, High Point, NC, USA) supplemented with 10% FBS, 100 U/mL Penicillin, and 100 µg/mL Streptomycin. All cell lines were confirmed to be free of mycoplasma through IDEXX or with MycoStrip™—Mycoplasma Detection Kit (Invivogen, San Diego, CA, USA). The cells were used for experiments at passage ≤ 20.

### 2.2. Cell Viability Assays and Synergy Analysis

Cells were seeded in 96-well plates overnight and treated with drug(s) for 48 h. For IC50 analysis cells were treated with a range of drug concentrations and viability was assessed by CellTiter-Glo Cell Viability Assay (Promega, Madison, WI, USA) following the manufacturer’s protocol. Luminescence for drug-treated wells was normalized to that of vehicle (DMSO)-treated control wells and Graphpad Prism 10.5.0 was used to determine IC50 values. For synergy analysis, cells were treated with romidepsin (Selleck, Houston, TX, USA) (0, 1.25, 2.5, 5, or 10 nM) alone or in combination with Doxorubicin (Cayman Chemical Company, Ann Arbor, Michigan, MI, USA) (0, 62.5, 125, 250 nM) or Etoposide (Selleck; 0, 15, 30, 60 µM). Luminescence of treated cells was normalized to vehicle control. Synergy was assessed using SynergyFinder 3.0 with the Bliss independence model [18]. Each experiment was performed in triplicate and repeated for ≥3 independent experiments.

### 2.3. In Vitro Combination Treatment and Analysis

Cells were seeded in 96-well plates overnight and treated with drug(s) for 48 h. Treatment consisted of cells being treated with VDC (20 nM vincristine (Selleck), 100 nM doxorubicin (Cayman Chemical Company), and 100 µM cyclophosphamide (Selleck)), IE (400 µM Ifosfamide (Selleck), and 30 µM Etoposide (Selleck)) or vehicle (DMSO). At the same time as the addition of chemotherapy combinations, romidepsin was added to each group at varying concentrations (0, 1.25, 2.5, 5 nM). Viability was assessed using CellTiter-Glo Cell Viability Assay. Luminescence of each treated well was normalized to that of vehicle treated controls. Graphpad Prism was used to apply two-way ANOVA to determine statistical significance. Each experiment was performed in triplicate and repeated for ≥3 independent experiments.

### 2.4. Western Blotting and Densitometry Analysis

Cells were seeded overnight and treated with drug(s) for 24 or 48 h. Treatment consisted of vehicle (DMSO), 2.5 nM or 5 nM of romidepsin for the single agent experiments. For combination experiments cells were treated with vehicle (DMSO), 5 nM romidepsin, 125 nM doxorubicin, and/or 15 µM etoposide. Protein isolation, quantification, and expression analysis including all antibodies used were performed as previously described [16].

### 2.5. Incucyte Caspase 3 Assay

5000 ES cells were seeded per well in a 96-well plate overnight. Incucyte^®^ Caspase-3/7 Dye and Nuclight Rapid Red Dye (Sartorius, Gottingen, Germany) were diluted in growth medium; medium was aspirated from the cells and the medium containing the Caspase 3/7 and Rapid Red Dyes were added to a final concentration of 1:1000 and 1:500, respectively. Drug treatments were added at the time of adding the Incucyte dyes. Cells were imaged every 12 h at 10×, and fluorescence quantification was analyzed using the Incucyte 2024A Basic Analyzer software.

### 2.6. In Vivo Experiments

2 × 10^6^ A673 cells were injected subcutaneously into female young adult athymic nude mice (The Jackson Laboratory, Bar Harbor, ME, USA). Once the average tumor volume reached 100–200 mm^3^ mice were evenly distributed to treatment groups to standardize average tumor volumes across groups. Treatment began immediately following grouping. Mice were treated by intraperitoneal injection (i.p.) with vehicle (PBS), romidepsin (2 mg/kg; twice per week), IE (90 mg/kg ifosfamide, 120 mg/kg etoposide; days 1–3 per cycle) [19], or a combination of romidepsin and IE. Tumor volumes, body weights, and body condition scores were measured ≥2 times per week. Mice were euthanized once tumors reached a size of 2000 mm^3^, reached ≥20 mm in any direction, or reached an ulceration score of 2 following approved Institutional Animal Care and Use Committee (IACUC) protocol guidelines. A total of 10 mice were allocated to each treatment group per experimental replicate. Mice which did not form tumors (3), had a drug treatment deviation (1), or were euthanized due to unrelated medical concerns (1), were excluded from the experiment. Mice were treated with two 21-day cycles of drug(s); following the end of treatment the remaining mice continued to be monitored for survival analysis.

## 3. Results

### 3.1. Romidepsin Decreases Cell Viability and Decreases Expression of Proteins Involved in DNA Damage Repair in Ewing Sarcoma Cells In Vitro

**Figure 1:**
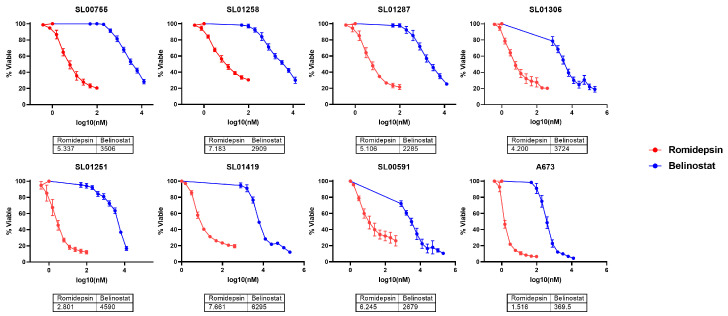
Ewing sarcoma cells are sensitive to HDAC inhibition. Ewing sarcoma patient derived cell lines were treated with HDAC inhibitors (Romidepsin or Belinostat) for 48 h. Viability was measured with CellTiter-Glo 2.0 and IC50s were calculated using the Absolute IC50 function in GraphPad Prism.

**Figure 2:**
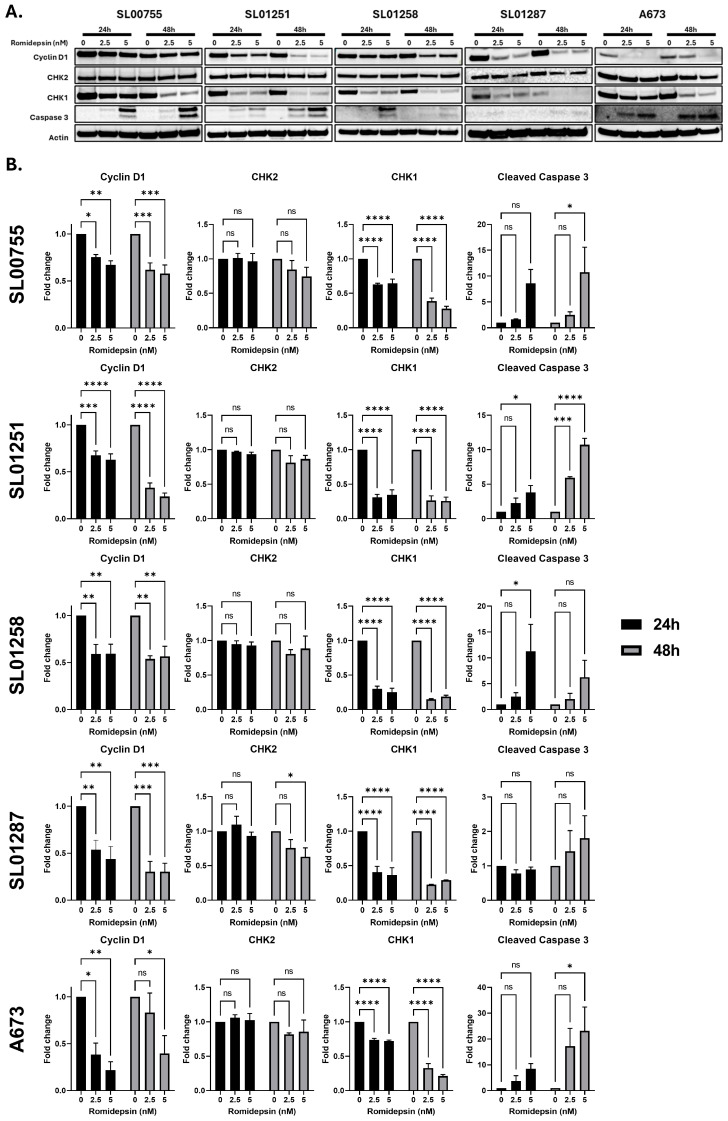
Romidepsin decreases expression of proteins involved in cell cycle progression and DNA damage repair. (**A**,**B**) Ewing sarcoma patient derived cell lines were treated with Romidepsin (0, 2.5 or 5 nM) for 24 or 48 h and protein expression was determined by Western blotting with the indicated antibodies (**A**). Signal intensities were quantified using AzureSpot 2.2.167 software to select bands and subtract background. Intensities were normalized to ß-Actin and fold changes were calculated for each drug treated sample over vehicle treated. Two-way ANOVA with Dunnett’s multiple comparisons test indicates significance, ns: not significant, *p* < 0.05 *, *p* < 0.01 **, *p* < 0.001 ***, *p* < 0.0001 **** (**B**). The uncropped bolts are shown in Supplementary Materials.

The HDAC inhibitors romidepsin and belinostat were screened for efficacy against a panel of ES patient derived cell lines. All ES cell lines screened were sensitive to both inhibitors at clinically relevant concentrations (Figure 1) [20]. Romidepsin is a selective HDAC 1/2 inhibitor and belinostat is a pan-HDAC inhibitor. Due to the strong efficacy of romidepsin against ES cells and the specificity for HDAC2, known to be overexpressed in ES tumors [16], the mechanism of romidepsin in ES cells was evaluated. A panel of ES cell lines was treated with romidepsin, at concentrations well below clinical relevance (2.5 or 5 nM), for 24 or 48 h and harvested for protein expression analysis. In all cell lines tested, expression of Cyclin D1 and CHK1 were significantly decreased (Figure 2 and Figure S2); with 5 nM romidepsin at 48 h, Cyclin D1 was decreased with a fold change of 0.3–0.58 (*p* < 0.01 **), and CHK1 was decreased with a fold change of 0.19–0.29 (*p* < 0.0001 ****) across all cell lines tested. Cyclin D1 is involved in cell cycle progression [21] and CHK1 in DNA damage repair [22]; both of these proteins have been shown to be overexpressed in ES tumors [16], indicating that these pathways may be involved in the toxicity of romidepsin against ES cells. CHK2, which also plays a role in the DNA repair process [22] was not significantly decreased by romidepsin with the exception of SL01287 cells where the fold change was 0.63 (*p* < 0.05 *) after 48 h of 5 nM romidepsin. Caspase 3 cleavage was increased, indicating induction of apoptosis (Figure 2 and Figure S2). Interestingly, the extent and timing of caspase 3 cleavage varied among cell lines; 5 nM romidepsin induced a fold change of 10.74–10.76 (*p* < 0.05) in SL00755 and SL01251 after 48 h of treatment, while the largest increase in SL01258 (11.3 fold change *p* < 0.05) was after only 24 h of 5 nM romidepsin treatment. A673 cells had the greatest increase in caspase 3 cleavage among all the cell lines tested with a 23.2-fold change (*p* < 0.05 *) after 48 h of 5 nM romidepsin.

### 3.2. Romidepsin Synergizes with Standard of Care Chemotherapeutics In Vitro

**Figure 3:**
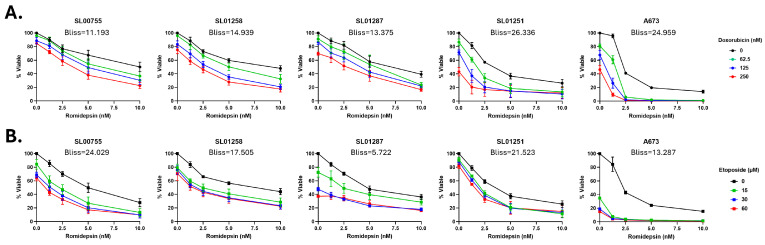
Romidepsin synergizes with standard of care chemotherapeutics in vitro. (**A**) Ewing sarcoma patient cells were treated with Romidepsin (0, 1.25, 2.5, 5, or 10 nM) alone or in combination with (**A**) Doxorubicin (0, 62.5, 125, 250 nM) or (**B**) Etoposide (0, 15, 30, 60 µM) for 48 h. Cell viability was measured using CellTiter-Glo 2.0; viability of drug treated cells was normalized to vehicle treated controls. Data shown represents the average of ≥3 experiments, and error bars are SEM. Bliss synergy scores were calculated using SynergyFinder 3.0. Bliss scores of −10 to 10 indicate an additive effect; scores of greater than 10 indicate a synergistic effect.

Because we know that romidepsin decreases CHK1, which is involved in cell cycle repair, we hypothesized that it would synergize with DNA damaging agents, including the chemotherapeutic agents used as standard of care for treatment of ES, doxorubicin, and etoposide. When romidepsin was combined with either doxorubicin or etoposide, all at clinically relevant concentrations, a synergistic response was observed (Bliss score > 10) (Figure 3). These data indicate that the addition of romidepsin to treatment may improve the efficacy of standard of care chemotherapy.

### 3.3. The Addition of Romidepsin to Standard of Care Chemotherapy Combinations Significantly Decreases ES Cell Viability

**Figure 4:**
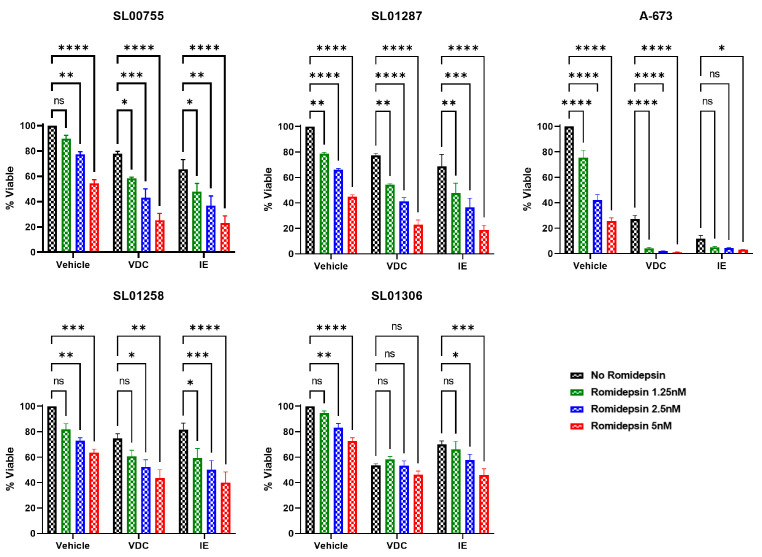
Romidepsin increases the efficacy of standard of care chemotherapy in vitro. Ewing sarcoma patient cells were treated with Romidepsin, standard of care chemotherapy combinations (VDC or IE), or a combination of Romidepsin with chemotherapy. Cell viability was measured following 48 h of treatment using CellTiter-Glo 2.0; viability of drug treated cells was normalized to vehicle only treated controls. Data shown represents the average of ≥3 experiments and error bars are SEM. Two-way ANOVA with Dunnett’s multiple comparisons test indicates significance, ns: not significant, *p* < 0.05 *, *p* < 0.01 **, *p* < 0.001 ***, *p* < 0.0001 ****. Drug concentrations: VDC—20 nM Vincristine, 100 nM Doxorubicin, 100 µM Cyclophosphamide; IE—400 µM Ifosfamide, 30 µM Etoposide.

The standard of care treatment for upfront ES includes alternating cycles of vincristine, doxorubicin, and cyclophosphamide (VDC) and ifosfamide and etoposide (IE). To test the efficacy of romidepsin with standard of care chemotherapy, a panel of ES cell lines was treated with VDC, IE, or no chemotherapy combined with a range of romidepsin concentrations (0–5 nM). A significant decrease in cell viability was observed when romidepsin was added to the IE treated cells compared to that of cells treated with IE alone in all cell lines tested (Figure 4). The same was true for cells treated with VDC, with the exception of SL01306. The observation that romidepsin increases efficacy of standard of care in vitro indicates that this may be a potential treatment strategy to improve patient outcomes.

### 3.4. The Combination of Romidepsin with Chemotherapeutics Leads to an Accumulation of DNA Damage in ES Cells

**Figure 5:**
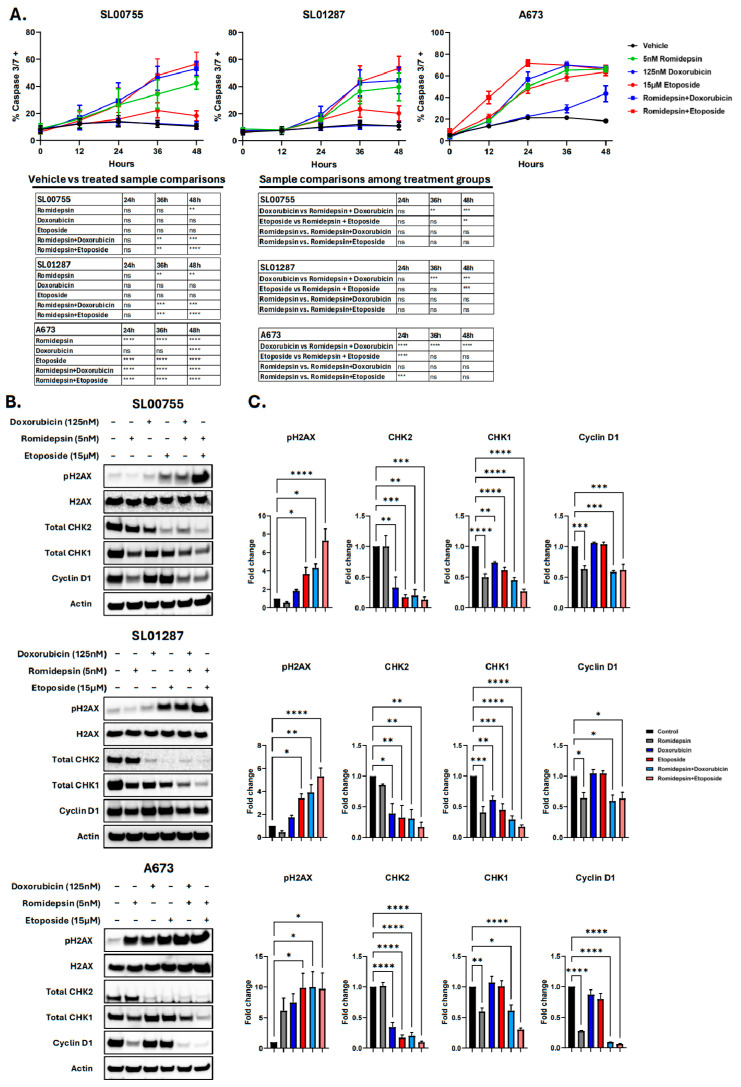
Romidepsin combined with chemotherapeutics leads to an accumulation of DNA damage. (**A**) ES cells were seeded and Incucyte Caspase 3/7 and Nuclight Rapid Red dyes were added at the time of treatment with romidepsin alone or in combination with chemotherapeutics. Cells were imaged for 48 h by an Incucyte S3 live cell imaging system and quantification of red and green fluorescence was performed using the Incucyte^®^ 2024A Basic Anlayzer software. Two-way ANOVA with Dunnett’s (vehicle vs. treated) or Tukey’s (treatment group comparisons) multiple comparisons tests indicate significance. (**B**,**C**) ES cell lines were treated with Romidepsin alone or in combination with standard of care chemotherapeutics at the indicated concentrations for 24 h and protein expression was determined by Western blotting with the indicated antibodies (**B**). Signal intensities were quantified using AzureSpot software to select bands and subtract background. Intensities were normalized to ß-Actin (pH2AX was normalized to total H2AX) and fold changes were calculated for each drug treated sample over vehicle treated. Two-way ANOVA with Dunnett’s multiple comparisons test indicates significance, ns: not significant, *p* < 0.05 *, *p* < 0.01 **, *p* < 0.001 ***, *p* < 0.0001 **** (**C**). The uncropped bolts are shown in Supplementary Materials.

To investigate the induction of apoptosis in response to the combination of romidepsin and SOC, cells were treated, and apoptosis was measured by Incucyte^®^ Caspase-3/7 Dye. In all three cell lines tested there was a significant induction of apoptosis in the combination treatment groups by 36 h of treatment, with the greatest increase being observed in response to the combination treatments (Figure 5A). Interestingly, SL01287 and SL00755 cells treated with the combination treatments had significantly more caspase 3/7+ cells compared to those treated with either doxorubicin or etoposide alone at the 48 h timepoint; the same was true for A673 cells at the 24 h timepoint. This indicates that the addition of romidepsin significantly improves the apoptotic effect of the SOC agents. To further investigate the mechanism of synergy observed between romidepsin and chemotherapeutic agents, cells were treated with either romidepsin, doxorubicin, or etoposide, alone or in combination, for 24 h and expression of proteins involved in DNA damage/repair and cell cycle were analyzed (Figure 5B,C and Figure S3). We hypothesized that the combination(s) of chemotherapeutics with romidepsin would lead to a decrease in DNA damage repair proteins (CHK1/2) and therefore an increase in DNA damage (pH2AX). Romidepsin treatment alone led to a decrease in CHK1 (0.41–0.60 fold change, *p* < 0.01) in all cell lines tested; doxorubicin and etoposide single agents also led to decreases in CHK1 in the SL00755 and SL01287 cells lines (fold changes 0.61–0.74 for doxorubicin (*p* < 0.01), and 0.44–0.61 for etoposide (*p* < 0.001)), though CHK1 was not decreased in A673 cells by the single agent chemotherapeutics. The combination treatments led to a significant decrease in CHK1 in all cell lines tested with a fold change of 0.29–0.61 (*p* < 0.05) for the romidepsin + doxorubicin groups, and 0.17–0.30 (*p* < 0.0001) for the romidepsin + etoposide groups, which was a greater decrease than what was observed with the single agent chemotherapy treatments. CHK2 was also decreased in all cell lines tested by single agent chemotherapeutics with a fold change of 0.33–0.39 (*p* < 0.05) in the doxorubicin treated cells and 0.17–0.32 (*p* < 0.01) in the etoposide treated cells. The combination treatments led to an even greater decrease with a fold change of 0.2–0.34 (*p* < 0.01) in the romidepsin + doxorubicin treated group and 0.10–0.17 (*p* < 0.01) in the romidepsin + etoposide treated groups. Further, we assessed the level of DNA damage by pH2AX expression. The only single agent treatment that led to a significant increase in pH2AX expression was etoposide with a fold change of 3.68–9.85 (*p* < 0.05). Both combination treatments led to a significant increase in pH2AX in all cell lines tested. The combination of romidepsin + doxorubicin led to a fold change of 3.91–9.99 (*p* < 0.05); the combination of romidepsin + etoposide led to a fold change of 5.29–9.70 (*p* < 0.05). Cyclin D1 expression was also assessed to investigate changes in cell cycle in response to the combination treatments. Romidepsin alone as well as in combination with doxorubicin or etoposide led to significant decreases in Cyclin D1 expression. Romidepsin alone decreased Cyclin D1 by a fold change of 0.27–0.64 (*p* < 0.05); the combination treatments led to a decrease of 0.10–0.59 (*p* < 0.05) and 0.06–0.62 (*p* < 0.05) in the romidepsin + doxorubicin and romidepsin + etoposide treated cells, respectively. These data indicate that the combination of romidepsin with DNA damaging chemotherapeutics leads to a decrease in proteins involved in cell cycle progression and DNA damage repair, thus leading to an accumulation of DNA damage. This suggests that the mechanism of synergy between these agents is likely due to romidepsin decreasing expression of proteins involved in the DNA repair mechanism, leading to the inability of the cells to repair and survive the DNA damage being induced by the chemotherapy agents.

### 3.5. The Addition of Romidepsin to IE Decreases Tumor Burden in a Xenograft Model

**Figure 6:**
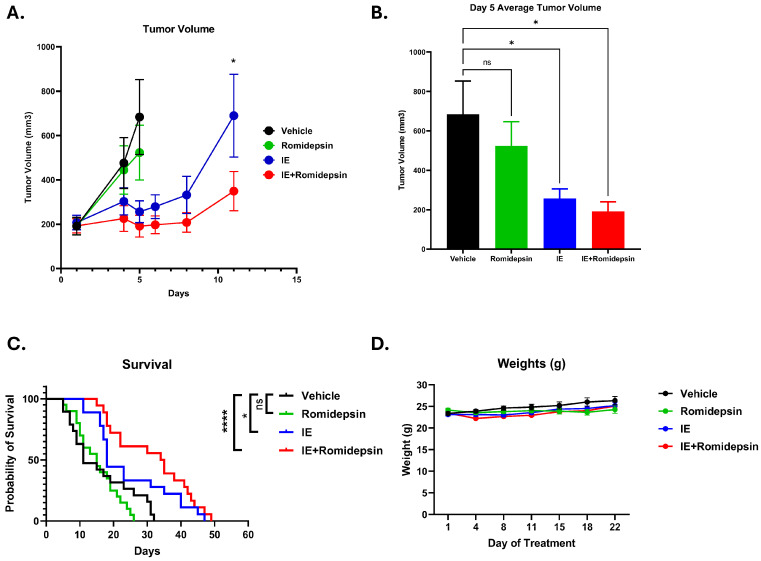
The addition of romidepsin to IE decreases tumor burden in a xenograft model. 2 × 10^6^ A673 cells were injected subcutaneously into mice. Once tumors were palpable, the mice were divided into groups and treated with two 21-day cycles of vehicle control (DMSO), romidepsin (2 mg/kg; twice per week), IE (90 mg/kg ifosfamide, 120 mg/kg etoposide; days 1–3 per cycle), or a combination of romidepsin and IE. Vehicle n = 19, romidepsin n = 20, IE n = 18, IE + romidepsin n = 18. (**A**) Average tumor volume over time. Two-way ANOVA indicates significance (*p* < 0.05 *) between the IE group and the IE + romidepsin group at day 11. (**B**) Average tumor volume on day 5 of treatment. One-way ANOVA with Tukey’s multiple comparisons test indicates significance (*p* < 0.05 *). (**C**) Survival curve, log-rank was test used to test for statistical differences, ns: not significant, *p* < 0.05 *, *p* < 0.0001 ****. (**D**) Average mouse weight for each group throughout the first cycle of treatment.

In vitro, we have observed that romidepsin synergizes with chemotherapeutics and increases the efficacy of chemotherapy combinations; therefore, we hypothesized that treatment of mice bearing subcutaneous ES tumors with a combination of romidepsin and chemotherapy would prolong survival compared to that of either of the drug(s) alone. Mice were injected with A673 cells subcutaneously and once tumors formed (100–200 mm^3^ average volume) they were evenly distributed to groups to ensure equal average tumor volumes. The mice were then treated with either vehicle, romidepsin (2 mk/kg twice weekly), IE (90 mg/kg ifosfamide, 12 mg/kg etoposide; days 1–3 per 21 d cycle) or the combination of IE + romidepsin. Groups treated with VDC, alone or in combination with romidepsin showed no significant differences in tumor volume or survival (Figure S1). On day 5, after the start of treatment with vehicle, romidepsin, IE, or IE + romidepsin, at least one mouse from the vehicle and romidepsin single agent groups was euthanized due to tumor burden. At that point, tracking of average tumor volume for those groups was stopped (Figure 6A). At day 5, there was no difference observed in tumor volume in the romidepsin group compared to vehicle (Figure 6B); however, there was a significant difference in volume in the IE and IE + romidepsin groups compared to vehicle (Figure 6B). The first mouse in the IE treatment group was euthanized on day 11 therefore average tumor volume tracking for both the IE and IE + romidepsin groups stopped at this timepoint. On day 11, there was a significant decrease in tumor volume in the IE + romidepsin group compared to the IE alone treatment group (Figure 6A). Survival analysis revealed a significant increase in survival in the IE and IE + romidepsin groups compared to vehicle, though no significant increase was observed in the IE + romidepsin group compared to IE alone (Figure 6C). There was no significant difference in survival between the vehicle and romidepsin treatment groups. No decline in body weight, body condition, activity, or appearance was observed in any group for the duration of the experiment indicating that the combination of IE + romidepsin may be well tolerated (Figure 6D).

## 4. Discussion

The observation that HDAC2, which is known to play an important role in tumor development and progression, is overexpressed in ES makes it an attractive therapeutic target [12]. Previous studies from our lab, as well as others, have shown efficacy of various HDAC inhibitors in vitro and/or in vivo [15,16]. HDAC inhibitors are clinically available, though none are approved for use in pediatric sarcomas [17,23]. Through this study, we have shown that romidepsin, a specific HDAC1/2 inhibitor, is effective against ES cells, synergizes with and improves efficacy of standard of care chemotherapeutics, and when combined with chemotherapy agents, leads to an accumulation of DNA damage in vitro. Further, the combination of romidepsin with IE leads to a significant reduction in tumor burden in vivo. These observations support the idea that the addition of HDAC inhibition, specifically romidepsin, to standard of care treatment may be a therapeutic option for ES patients.

A variety of HDAC inhibitors have been developed with various specificities. Pan-HDAC inhibitors, including Panobinostat, belinostat, and vorinostat, target multiple classes of HDACs, while others were developed to target specific HDACs or specific classes of HDACs [17,24]. Some examples of these specific HDAC inhibitors include romidepsin (class 1 HDACs), Ricolinostat (HDAC6), and Entinostat (HDAC 1&3) [25,26]. It has been shown that class 1 HDACs play a role in ES pathogenesis and that HDAC2 is overexpressed in ES patient samples, which suggests that, although pan-HDAC inhibitors may also be effective, specific class 1 HDAC inhibitors, such as romidepsin, may be a more specific approach to target ES cells. Further, a recent report has shown that HDAC inhibitors, including romidepsin, decrease expression of proteins related to DNA replication stress response, including CHK1 specifically. This report discussed that these effects of HDAC inhibitors may aid in sensitizing ES cells to other therapies, though it was not investigated in that study [27]. Our data is consistent in that we observed a significant decrease in CHK1 expression by romidepsin in all cell lines tested, and we further show that the combination of romidepsin with SOC leads to a significant increase in DNA damage, supporting the idea that HDAC inhibition sensitizes ES cells to SOC agents.

The FDA has approved HDAC inhibitors for use in various heme malignancies, and romidepsin is approved for treatment of cutaneous T-cell lymphoma. While there have been several clinical trials using HDAC inhibitors in solid tumors, as single agents there have not been many encouraging results reported [28,29]. A phase 1/2 trial investigating vorinostat in pediatric patients with relapsed solid tumor, lymphoma, or leukemia reported a best overall response (combining PR and SD) of 22%, with no CR observed [30]. A trial investigating romidepsin in renal cell carcinoma observed only a 7% overall response rate [31]. It is interesting that though in vitro data of efficacy of HDAC inhibitors against ES is encouraging, single agent efficacy has not been observed thus far in either mouse models or clinical trials [15]. Consistently, we observed no effect of romidepsin as a single agent in our mouse model. This highlights the need for combinatory therapy, which we have shown has a much greater effect in the reduction in tumor volume.

For our in vivo experiment, our dosing strategy consisted of mice being treated with IE on days 1–3 of each 21-day cycle and romidepsin being given twice weekly. Interestingly, around day 22 of the survival curve we see a large difference in survival between the IE alone and IE + romidepsin groups; this timepoint would be at the start of cycle 2. This may indicate that while the drugs (IE + romidepsin) were being given together, there was a real synergistic effect between the drugs. However, once the IE was stopped and the mice were only receiving romidepsin that synergy was lost, which is why we did not observe a significant increase in survival. This suggests that altering the dosing schedule may further improve the survival of the ES tumor bearing mice, which would be the goal of future studies.

*STAG2* and *TP53* are two of the most common genetic mutations observed in ES and have been shown to be co-associated in highly aggressive ES tumors [32]. A673 cells are wild-type for *STAG2* and harbor a p.A119fs *TP53* mutation. Although it has been shown that the probability of survival is lower for patients with a *TP53* mutation compared to wild-type, the probability of survival for patients with both a *TP53* and *STAG2* mutation is even lower. This may indicate that these genetic changes play a role in treatment response; therefore, another goal of future studies would be to test these drug combinations in mouse models generated from a variety of cell lines with known genetic backgrounds, specifically those with highly aggressive mutational backgrounds. This would aid in determining which patient populations would benefit most from this therapeutic combination.

## 5. Conclusions

Overall, this study shows the efficacy of HDAC 1/2 inhibition, specifically by romidepsin, in ES cells in vitro. Further, we show that romidepsin decreases the expression of proteins involved in DNA damage response and synergizes with standard of care drugs known to induce DNA damage. Finally, we show the efficacy of romidepsin in combination with IE in the reduction in tumor volume in ES mouse models. We believe that this study provides evidence for the potential of romidepsin to be used in combination with standard of care therapy for the treatment of Ewing sarcoma.

## Data Availability

The data presented in these studies are available upon request from the corresponding author.

## Supplementary Materials

The following supporting information can be downloaded at: https://www.mdpi.com/article/10.3390/cancers17244018/s1, Figure S1: In vivo model of treatment with VDC alone and in combination with romidepsin. Figure S2: Original blots related to Figure 2. Figure S3: Original blots related to Figure 5.
