# Supplementary material for: In Vitro and In Vivo Efficacy of Romidepsin Alone and in Addition to Standard of Care for Treatment of Ewing Sarcoma
Source: Cancers (Basel). 2025 Dec 17;17(24):4018. doi: 10.3390/cancers17244018 (PMC12730613; doi:10.3390/cancers17244018)

Figure S1. *In vivo* model of treatment with VDC alone and in combination with romidepsin.

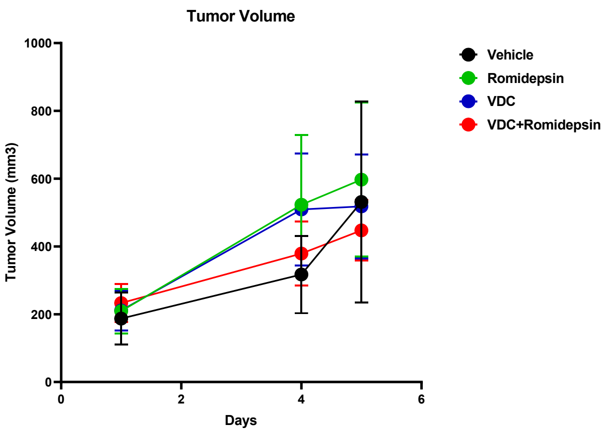

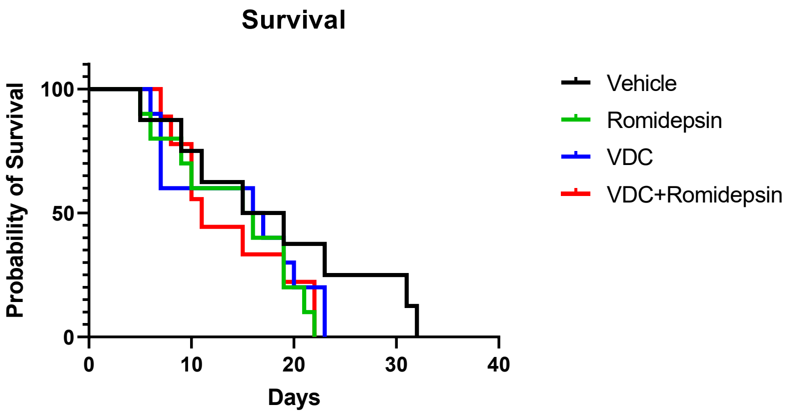

Figure S1. *In vivo* model of treatment with VDC alone and in combination with romidepsin. $2x10^6$ A673 cells were injected subcutaneously into mice. Once tumors were palpable the mice were divided into groups and treated with up to two 21-day cycles of vehicle control (DMSO), romidepsin (2mg/kg; twice per week), VDC (0.5mg/kg vincristine, 1.25mg/kg doxorubicin, 20mg/kg cyclophosphamide; day 1 per cycle), or the combination of VDC and romidepsin. N≥8 for each group. (A) Average tumor volume over time. (B) Survival curve.

Figure S2. Original blots related to Fig. 2.

SL00755

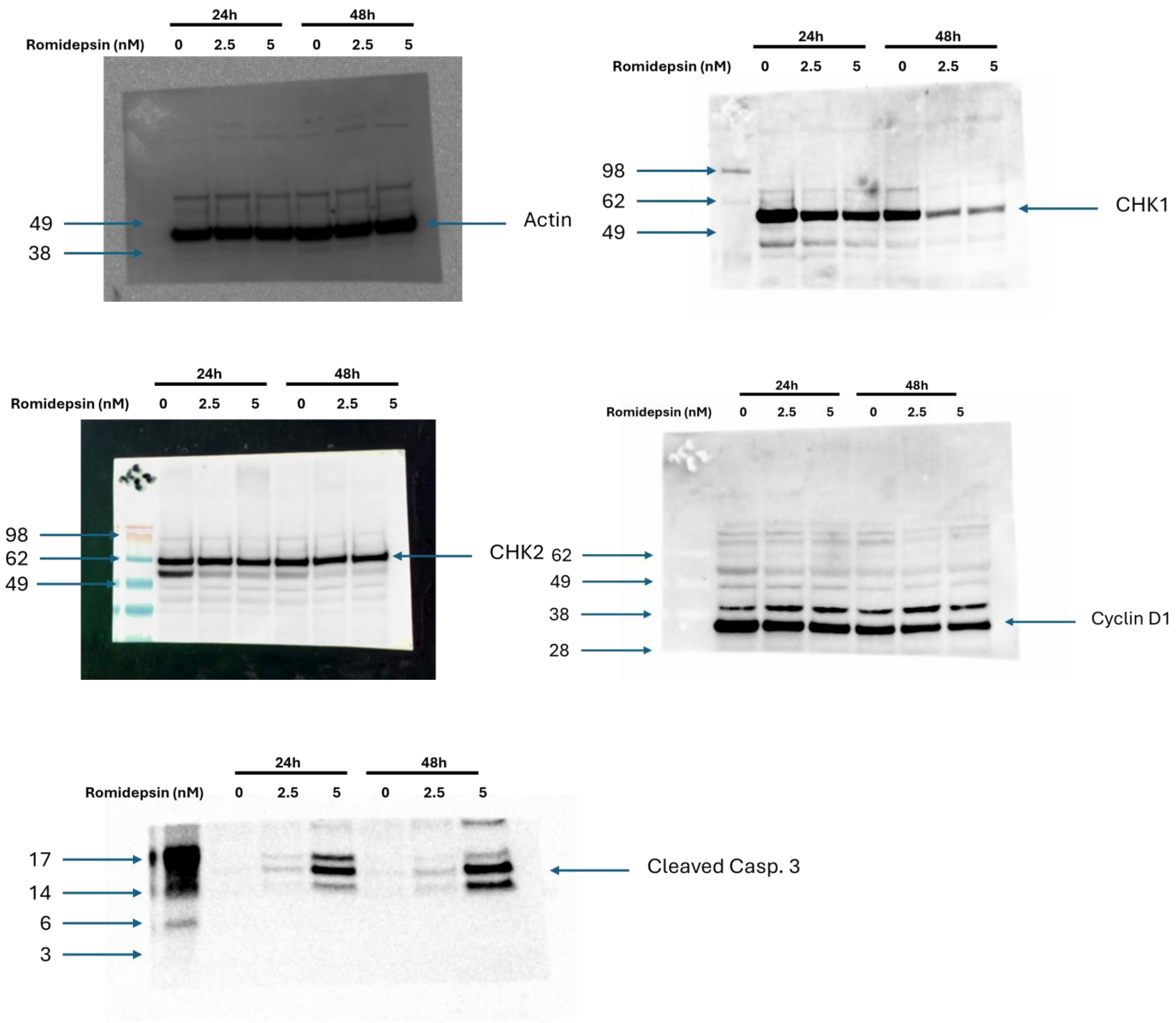

Figure S2. Original blots related to Fig. 2.

SL01251

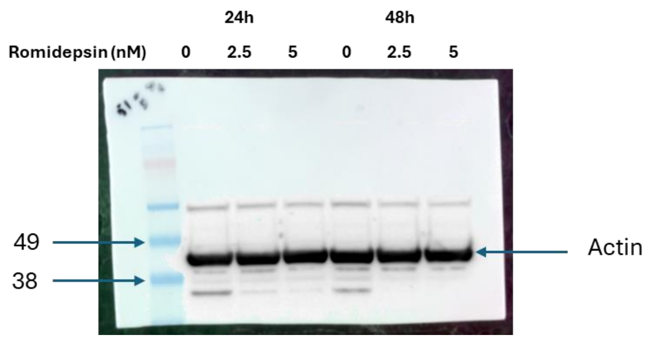

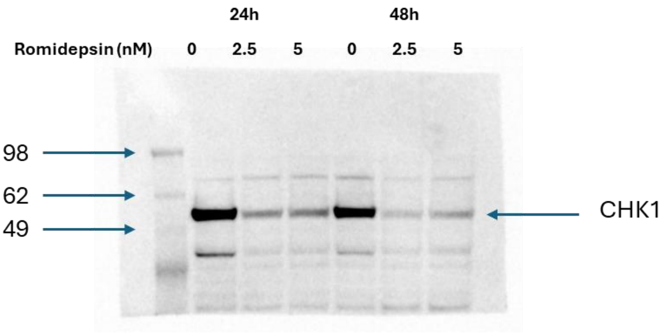

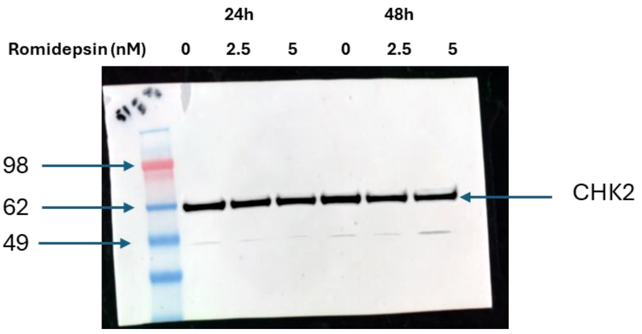

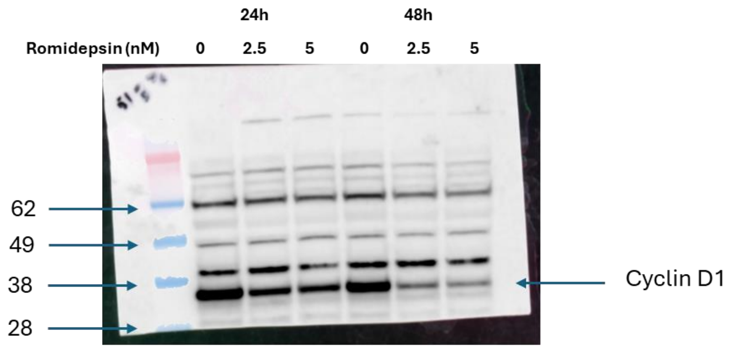

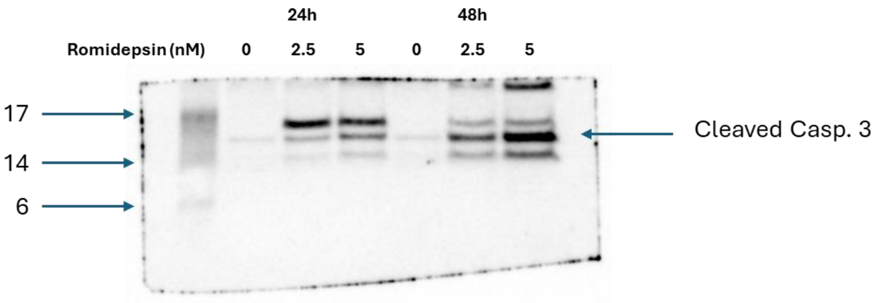

Figure S2. Original blots related to Fig. 2.

SL01258

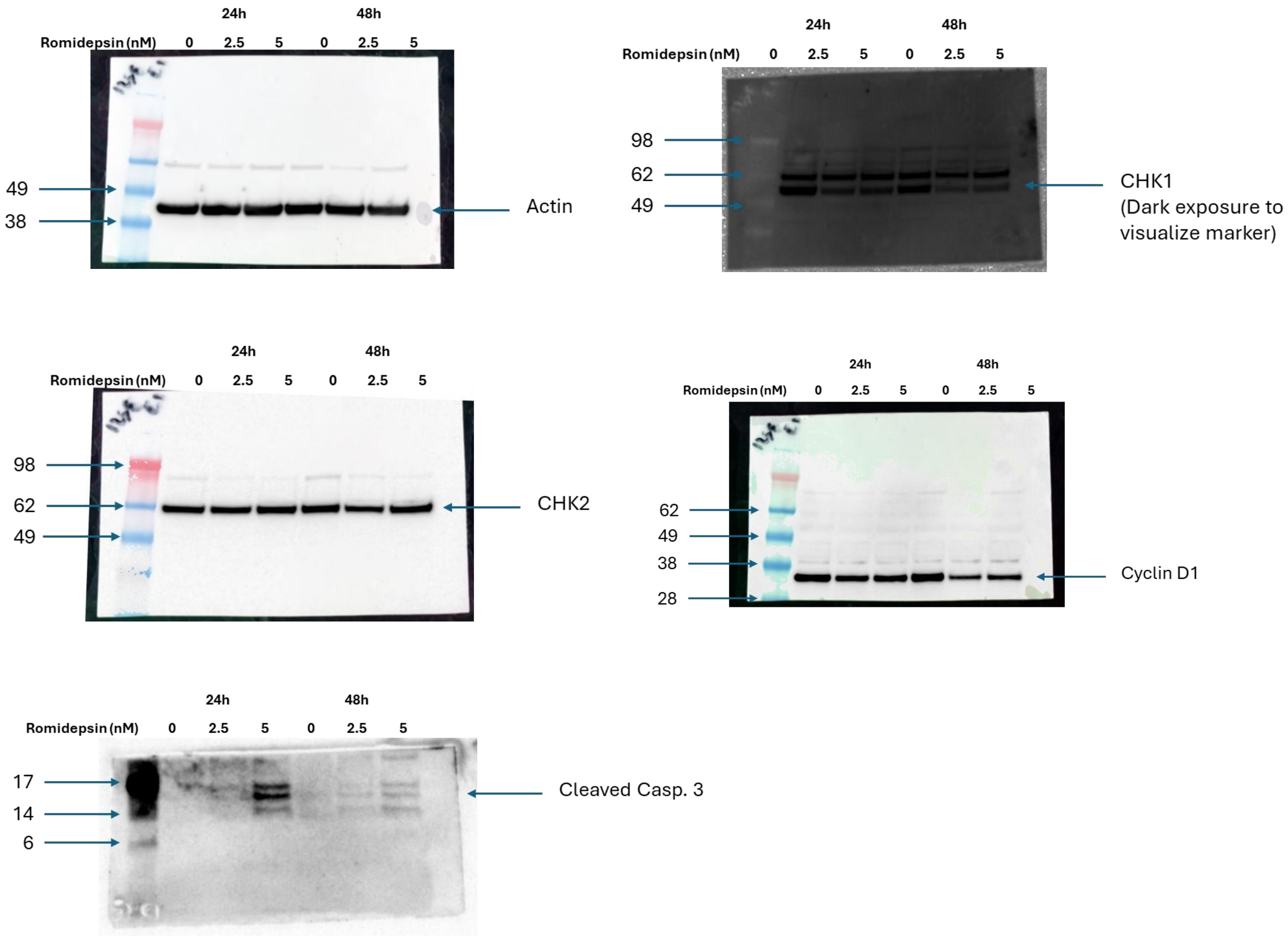

Figure S2. Original blots related to Fig. 2.

SL01287

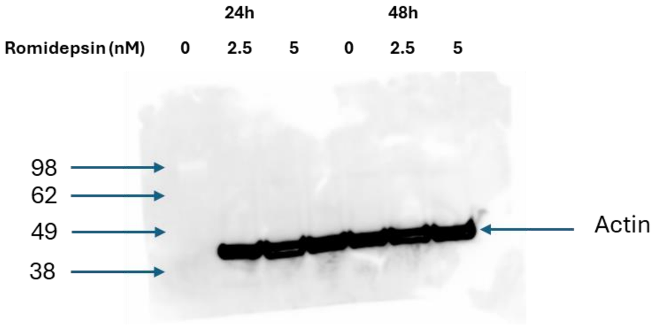

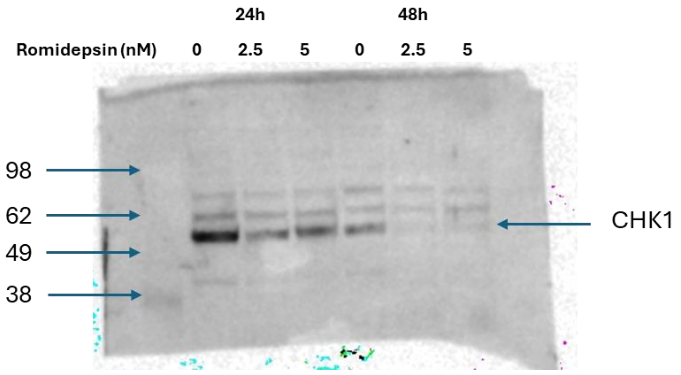

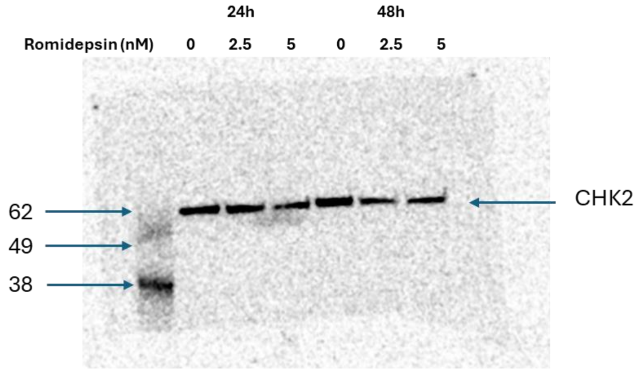

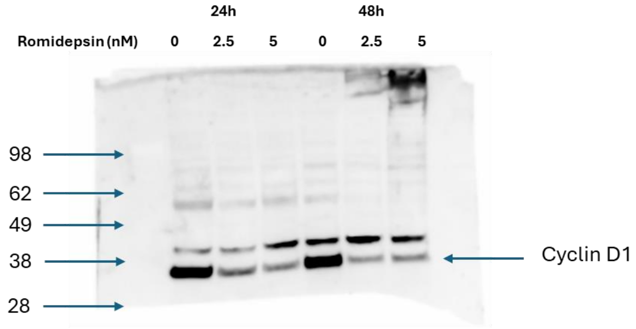

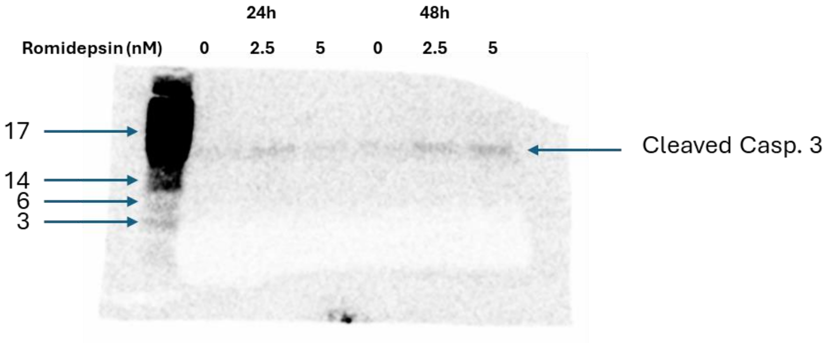

Figure S2. Original blots related to Fig. 2.

A673

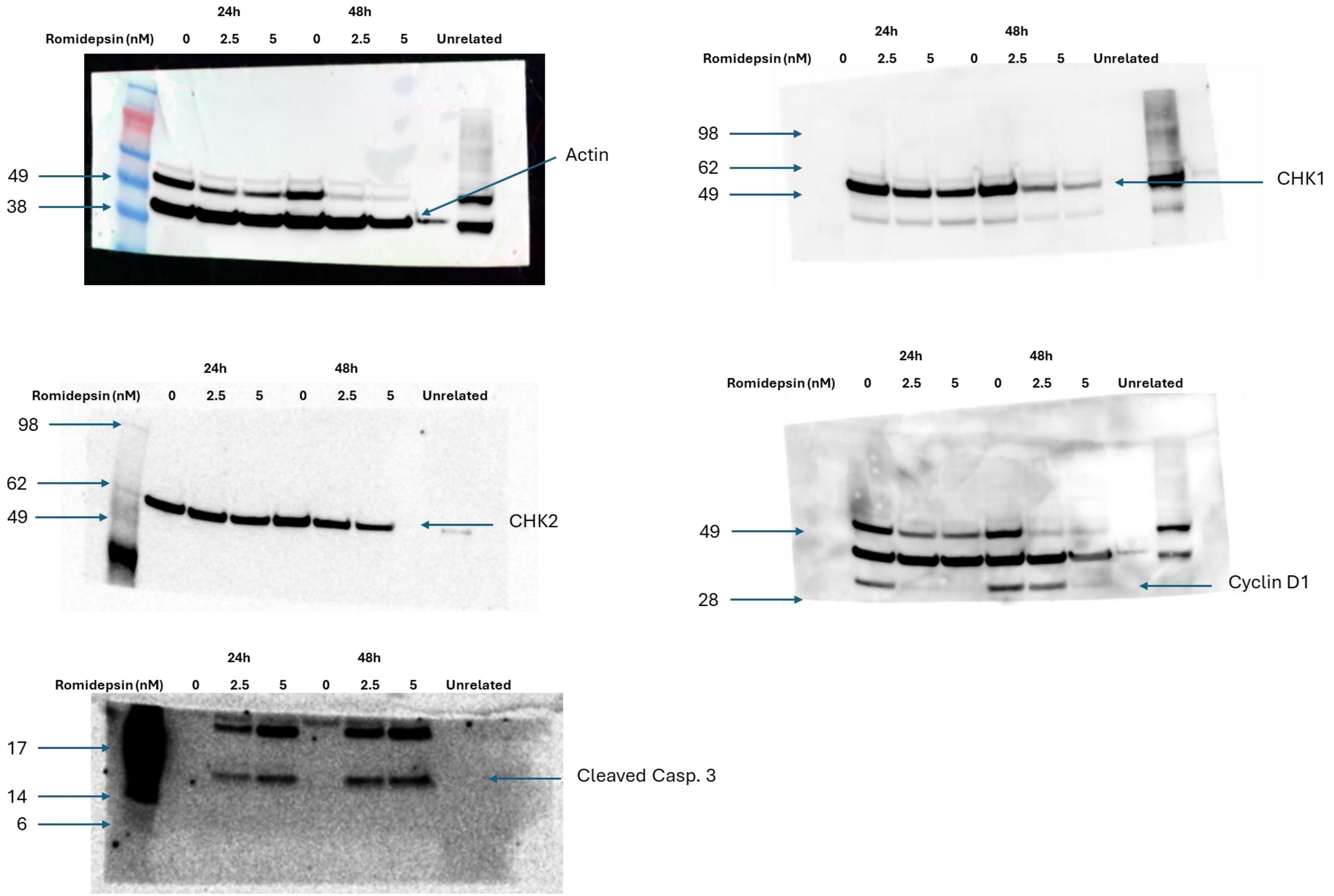

Figure S3. Original blots related to Fig. 5.

SL00755

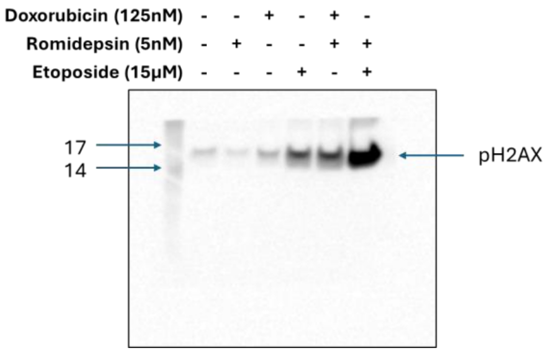

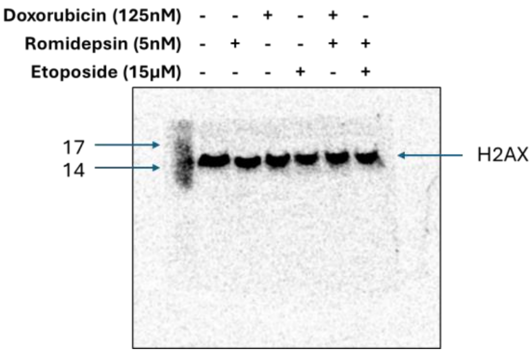

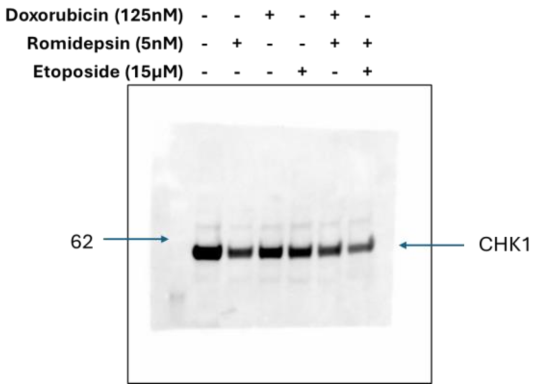

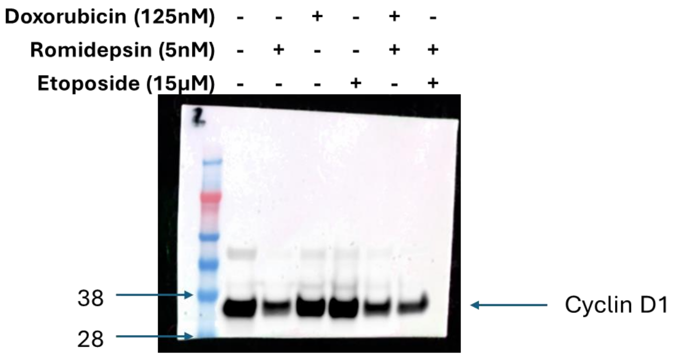

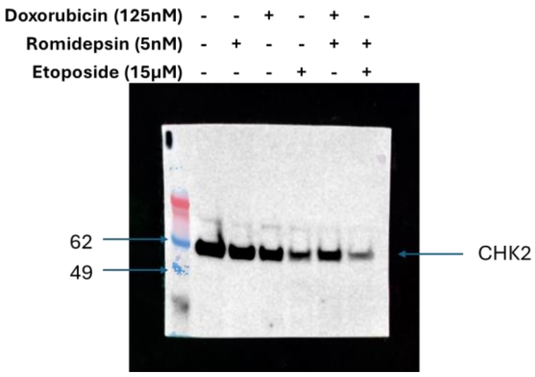

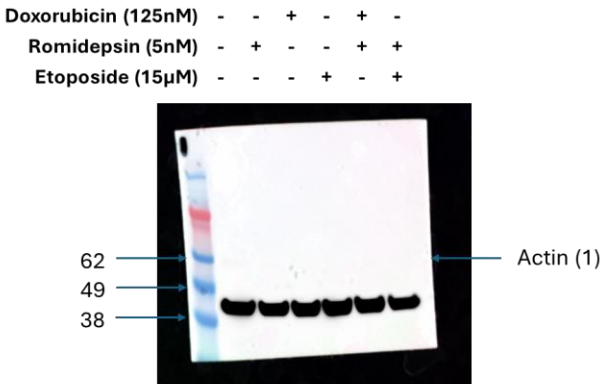

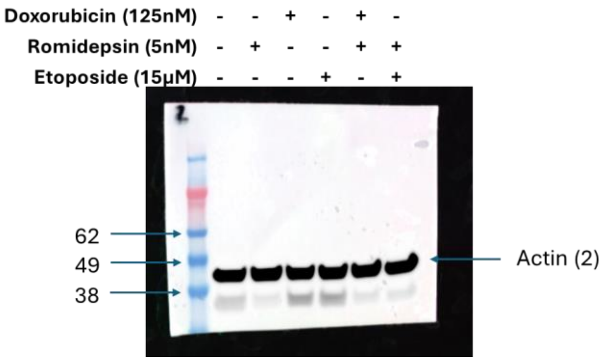

Figure S3. Original blots related to Fig. 5.

SL01287

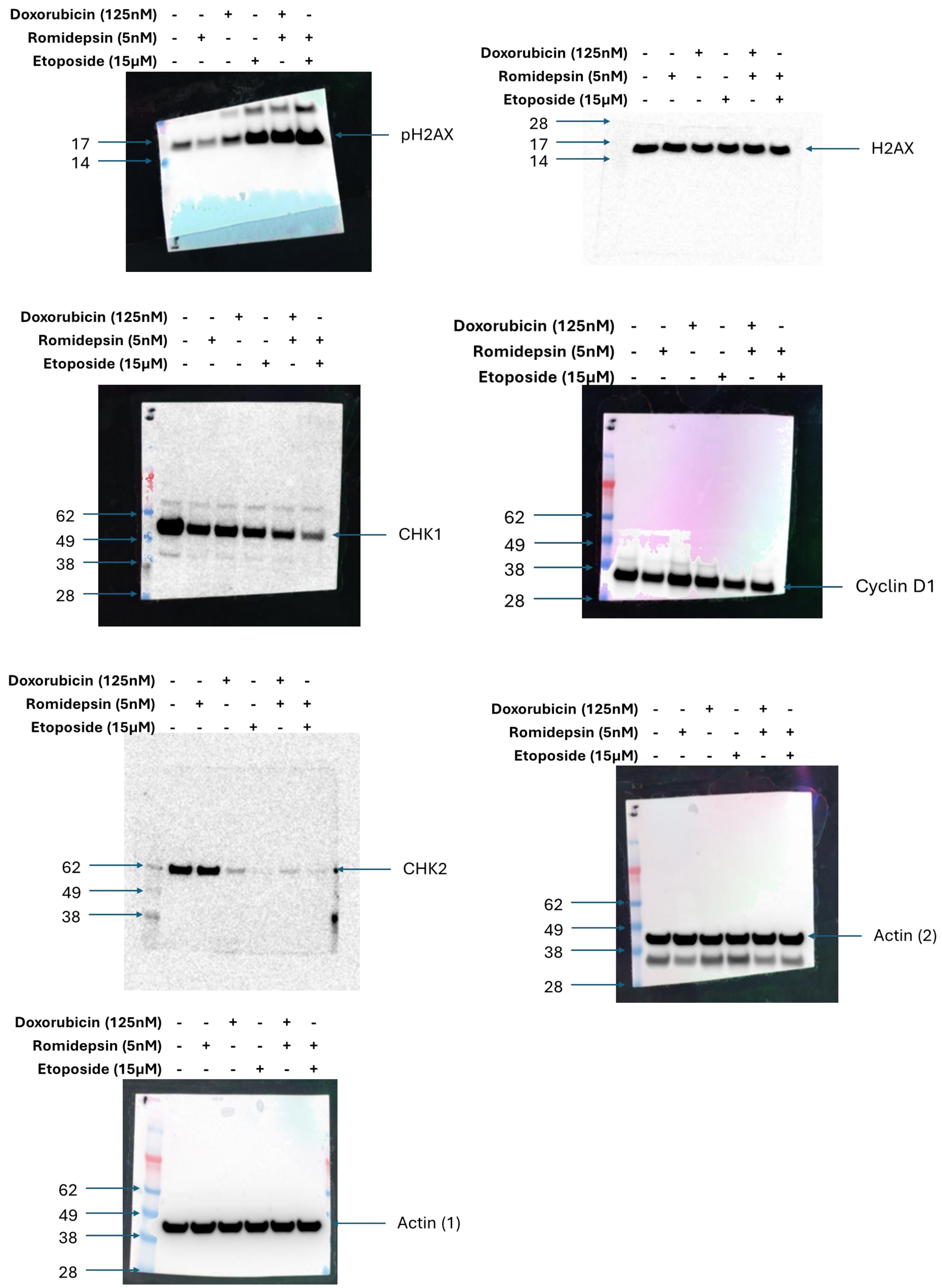

Figure S3. Original blots related to Fig. 5.

A673

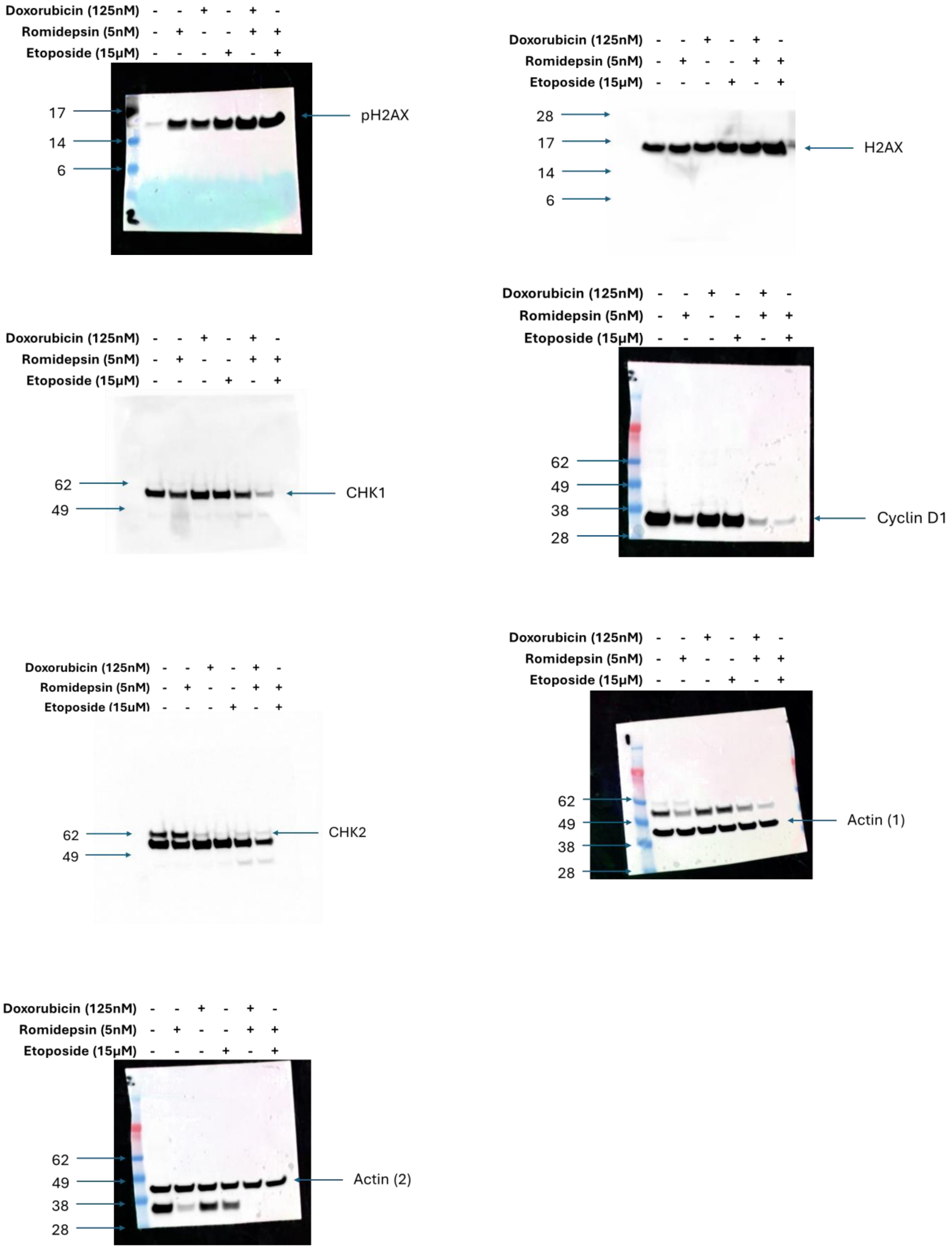

Supplement: Supplementary file 1 [file cancers-17-04018-s001.zip › cancers-4013303-supplementary.pdf]
